# Service-level barriers to and facilitators of accessibility to treatment for problematic alcohol use: a scoping review

**DOI:** 10.3389/fpubh.2023.1296239

**Published:** 2023-12-01

**Authors:** Dianna M. Wolfe, Brian Hutton, Kim Corace, Nathorn Chaiyakunapruk, Surachat Ngorsuraches, Surapon Nochaiwong, Justin Presseau, Alyssa Grant, Mackenzie Dowson, Amelia Palumbo, Kelly Suschinsky, Becky Skidmore, Mary Bartram, Gordon Garner, Lisha DiGioacchino, Andrew Pump, Brianne Peters, Sarah Konefal, Amy Porath Eves, Kednapa Thavorn

**Affiliations:** ^1^Ottawa Hospital Research Institute, Ottawa, ON, Canada; ^2^School of Epidemiology and Public Health, University of Ottawa, Ottawa, ON, Canada; ^3^Substance Use and Concurrent Disorders Program, Royal Ottawa Mental Health Centre, Ottawa, ON, Canada; ^4^Department of Psychiatry, Faculty of Medicine, University of Ottawa, Ottawa, ON, Canada; ^5^University of Ottawa Institute of Mental Health Research at the Royal, Ottawa, ON, Canada; ^6^Department of Pharmacotherapy, College of Pharmacy, University of Utah, Salt Lake City, UT, United States; ^7^Informatics, Decision Enhancement, and Analytics Sciences (IDEAS) Center, Veterans Affairs Salt Lake City Healthcare System, Salt Lake City, UT, United States; ^8^Harrison College of Pharmacy, Auburn University, Auburn, AL, United States; ^9^Department of Pharmaceutical Care, Pharmacoepidemiology and Statistics Research Center, Faculty of Pharmacy, Chiang Mai University, Chiang Mai, Thailand; ^10^Mental Health Commission of Canada, Ottawa, ON, Canada; ^11^School of Public Policy and Administration, Carleton University, Ottawa, ON, Canada; ^12^Community Addictions Peer Support Association, Ottawa, ON, Canada; ^13^Canadian Centre on Substance Use and Addiction, Ottawa, ON, Canada; ^14^Children’s Hospital of Eastern Ontario Research Institute, Ottawa, ON, Canada; ^15^Knowledge Institute on Child and Youth Mental Health and Addictions, Ottawa, ON, Canada

**Keywords:** problematic alcohol use, alcohol use disorder, addiction medicine, scoping review, barriers, treatment, review, knowledge synthesis

## Abstract

**Introduction:**

Services to treat problematic alcohol use (PAU) should be highly accessible to optimize treatment engagement. We conducted a scoping review to map characteristics of services for the treatment of PAU that have been reported in the literature to be barriers to or facilitators of access to treatment from the perspective of individuals with PAU.

**Methods:**

A protocol was developed *a priori*, registered, and published. We searched MEDLINE^®^, Embase, the Cochrane Library, and additional grey literature sources from 2010 to April 2022 to identify primary qualitative research and surveys of adults with current or past PAU requiring treatment that were designed to identify modifiable characteristics of PAU treatment services (including psychosocial and pharmacologic interventions) that were perceived to be barriers to or facilitators of access to treatment. Studies of concurrent PAU and other substance use disorders were excluded. Study selection was performed by multiple review team members. Emergent barriers were coded and mapped to the accessibility dimensions of the Levesque framework of healthcare access, then descriptively summarized.

**Results:**

One-hundred-and-nine included studies reported an extensive array of unique service-level barriers that could act alone or together to prevent treatment accessibility. These included but were not limited to lack of an obvious entry point, complexity of the care pathway, high financial cost, unacceptably long wait times, lack of geographically accessible treatment, inconvenient appointment hours, poor cultural/demographic sensitivity, lack of anonymity/privacy, lack of services to treat concurrent PAU and mental health problems.

**Discussion:**

Barriers generally aligned with recent reviews of the substance use disorder literature. Ranking of barriers may be explored in a future discrete choice experiment of PAU service users. The rich qualitative findings of this review may support the design of new or modification of existing services for people with PAU to improve accessibility.

**Systematic Review Registration:**

Open Science Framework doi: 10.17605/OSF.IO/S849R.

## 1 Introduction

Alcohol consumption is a leading cause of death and disability, associated with numerous dose-dependent negative physical and mental health problems (1, 2) that contribute to a high global burden of disease (2). Despite disabling consequences at both individual and population levels, rates of treatment seeking for alcohol use disorder (AUD) and problematic alcohol use (PAU)—hereafter simply “PAU”—are remarkably low, globally, and have potentially declined over time (3, 4). In 2020, the COVID-19 pandemic exposed gaps in the continuum of PAU treatment services, raising concerns that reductions in treatment availability (5–10) would further decrease treatment seeking and engagement. To mitigate pandemic-related barriers, treatment delivery was modified in many countries to embrace telehealth options (11–18). However, for some treatment seekers, telehealth was challenging and was itself a barrier to treatment engagement (17). Comprehensive knowledge of the barriers to PAU treatment is necessary to develop mitigation strategies to improve the accessibility of existing treatment services, to guide future policy reform, and to establish resiliency in treatment accessibility during future unanticipated service disruptions.

Many factors can impede help-seeking and engagement in treatment for substance use problems, including individual, social, and structural barriers. At the individual level, lack of awareness or acceptance of the severity of the substance use problem (4, 19), belief in capacity to self-manage (4, 19), negative attitude toward treatment from past experience (4, 19), and negative emotional states, such as depression (20), can decrease the motivation to seek treatment. Social barriers, such as stigmatization of people who disclose their challenges with PAU (4, 19, 20), normalization of drinking (21), and sociocultural and gender norms regarding treatment seeking (4) also lower treatment seeking rates. Structural barriers to treatment accessibility encompass both policy-level barriers (e.g., coverage of PAU treatment by government and/or private insurance) and service-level barriers (e.g., fragmented treatment pathways, lengthy waiting periods (22–26)). Service-level barriers can be wide ranging, including treatment seekers not knowing where to access care; lack of treatment services in a given region; lack of sufficient service supply, which leads to long wait times; inappropriateness of services for some demographics; and a limited range of treatment options. Accessibility may be challenging due to practical issues, such as inflexible or inconvenient appointment times, and problems with cost, transportation, or childcare. Service restrictions to certain demographics (e.g., men-only mutual aid groups) and lack of cultural sensitivity and safety in treatment programs (e.g., for Indigenous people and other ethno-cultural groups) can further exclude or dissuade treatment seekers. Behavior change theories suggest that environmental factors, such as service-level barriers, can decrease engagement in treatment, even when the necessary positive attitude toward the behavior (i.e., engagement in treatment), social factors, and the skills and capacity required to perform the behavior are all present (27, 28). Thus, for people with PAU, real or perceived service-level barriers decrease treatment engagement, even when motivation to change drinking behavior is high (29). Identifying modifiable treatment barriers at the service level is important for developing strategies to improve treatment uptake among people with PAU. To address this important objective, we addressed the following question using a scoping review approach:

What characteristics of services for the treatment of PAU have been identified in the literature as barriers to or facilitators of access to the services from the perspective of the individuals with PAU?

## 2 Methods and analysis

We used a scoping review approach to identify and map existing evidence related to the review question above (30). The scoping review was underpinned by the Arskey and O’Malley (31) framework and was guided by methodology provided by the Joanna Briggs Institute (JBI) (32) and other sources (33–35). Reporting has been guided by the Preferred Reporting Items for Systematic reviews and Meta-Analyses extension for Scoping Reviews (PRISMA-ScR) (36) (Supplementary Table 1), as well as the Enhancing Transparency in Reporting the Synthesis of Qualitative Research (ENTREQ) guidelines (37) (Supplementary Table 1). The methods reported below include amendments that were made during the scoping review process. For transparency, we describe these amendments along with rationale in Supplementary Table 2.

### 2.1 Protocol and registration

A review protocol was published (38) and registered with the Open Science Framework (doi:10.17605/OSF.IO/S849R).

### 2.2 Eligibility criteria

Review eligibility criteria were based upon the PCC (Participants – Concept – Context) framework (32).

#### 2.2.1 Participants

We included studies that enrolled service users, which were defined as adults (18+ years), with current or past PAU of any definition that requires/required treatment, as determined by the individual (i.e., treatment seeking) or another actor (e.g., study investigators, primary healthcare, criminal justice system). Subgroups of service users were of interest, including Indigenous peoples and other ethno-cultural groups, individuals with PAU and concurrent mental health conditions (“dual diagnoses”), sex and gender subgroups, older adults, and others. Because different factors may influence access to treatment for PAU, substance use disorders (SUDs), and mental health conditions, we excluded studies of (1) people with concurrent PAU and other SUDs, unless they also had a concurrent mental health condition; (2) mixed populations of (a) people with PAU and people with other SUDs and (b) people with and people without PAU, unless relevant findings were reported separately for the former. Studies of people with/without PAU were included if they assessed a PAU screening intervention. Studies including both service users and service providers were included if relevant findings specific to the service users were reported.

#### 2.2.2 Concept

- *Research design criteria*: We included qualitative and cross-sectional primary research studies designed to identify modifiable characteristics (i.e., can be altered to mitigate a barrier) of PAU services that service users perceived to be barriers to or facilitators of access to the services.

“Access” was restricted to the search for treatment and initiation of treatment (or screening), including referral to specialist care. We did not consider either the period between a recognized desire for treatment and the subsequent search for care or the continuation of care once it had been initiated to be part of access (39).

- *Intervention criteria*: Both psychosocial and pharmacologic interventions were of interest.

o Psychosocial: Screening for PAU, either standalone or in combination with brief intervention and/or referral (i.e., SBIRT), brief interventions, mutual aid groups (e.g., 12-step groups), self-help (e.g., online programs, books), cognitive behavioral therapy (CBT), motivational interviewing, contingency management, family-based therapy, mindfulness-based interventions, motivational enhancement therapy, community reinforcement therapy, etc.
o Pharmacologic: Naltrexone, acamprosate, disulfiram, gabapentin, topiramate, baclofen, ondansetron.

Screening and SBIRT for PAU were considered relevant treatment services because they are often the initial point of access with the treatment system. Treatment of acute withdrawal symptoms was not relevant unless concurrent with a relevant psychosocial or pharmacologic intervention.

- *Setting/context criteria*: Service delivery could occur in any context, such as clinical (e.g., emergency department (ED), primary care, specialist clinics, live-in/hospital) or non-clinical (e.g., workplace settings, police custody/detention facilities, college/university) settings, including both in-person and virtual (i.e., telephone, video) services.
- *Data criteria*: To be eligible, studies must have reported at least one potentially modifiable characteristic of a PAU service that was perceived to be a barrier or facilitator of accessibility (e.g., appointment hours, cost, location) and that was identified through qualitative data (interview or focus group) or survey data analysis. Any primary study design was relevant; however, routinely collected administrative data and data from patient medical records were excluded. Interview studies with fewer than 13 participants were excluded, unless reporting a claim of data saturation (40).
- Barriers related to service users’ capacities were relevant if they corresponded to modifiable service-level factors (e.g., service user inability to afford treatment corresponded to treatment cost as a service-level barrier). However, factors related to the service user (e.g., readiness to change, lack of recognition/acknowledgment of a problem), the healthcare system/government (e.g., lack of government insurance coverage), and society (e.g., stigma, social norms of drinking) were not relevant.
- Studies were excluded if they reported only characteristics of specific interventions that could not be generalized to other interventions (e.g., elements of the user interface of mobile apps).

#### 2.2.3 Context

We decided *a priori* to focus on literature published from 2010 to present (including pandemic-related studies), given that provision of PAU services has changed over time and these changes may have influenced the perceived barriers to and facilitators of access (e.g., increased provision of virtual care). All geographic areas were of interest; however, we limited inclusion to studies published in English and French due to time and resource constraints. We excluded conference abstracts, letters, and commentaries, given the low likelihood of sufficient reporting for our purposes. Systematic reviews, rapid reviews and scoping reviews were excluded.

### 2.3 Literature search

A pre-planned search approach was used, involving comprehensive search strategies to identify all available studies. Search strategies were developed by an experienced information specialist in consultation with the review team. Using the multifile option and deduplication tool available on the Ovid platform, we searched Ovid MEDLINE^®^ ALL, Embase Classic + Embase, APA PsycInfo, EBM Reviews - Cochrane Central Register of Controlled Trials, and EBM Reviews - Cochrane Database of Systematic Reviews. CINAHL was searched on Ebsco. The MEDLINE strategy was peer reviewed by a second senior information specialist using the PRESS Checklist (41). Results were limited to English or French and to the publication years 2010 to the present. All searches were executed on April 5, 2022.

The strategies utilized a combination of controlled vocabulary (e.g., “Alcohol-Related Disorders,” “Mental Health Services,” “Health Services Accessibility”) and keywords (e.g., “PAU,” “counselling,” “social values”). Vocabulary and syntax were adjusted across the databases. Where possible, animal-only records, opinion pieces, case reports, books and book chapters were removed. Specific details regarding the strategies appear in Supplementary Table 3.

A targeted grey literature search of COVID-19-related sites was conducted, including the Cochrane COVID-19 Study Register, COVID-END, L-OVE, UNCOVER, and the WHO COVID-19 Database.

### 2.4 Study selection

Records were downloaded and deduplicated using EndNote version 9.3.3 (Clarivate Analytics) and uploaded to DistillerSR^®^ online software where further duplicates were removed (DistillerSR. Version 2.38. Evidence Partners; 2022. https://www.evidencepartners.com). Two levels of screening were performed: citations included at initial screening of titles and abstracts were subsequently screened in full text. To calibrate the screening process, pilot testing of screening forms was conducted on batches of 50 citations at Level 1 and two citations at Level 2 until conflicts between reviewers were reduced to approximately 5% and reviewers were comfortable with the screening process.

To expedite screening, at Level 1, the artificial intelligence (AI) active-machine learning (AML) feature of DistillerSR^®^ was used to prioritize screening of citations most likely to be relevant. A set of 200 citations that included both included and excluded studies was initially screened by two human reviewers to train the AML tool’s classification algorithm. Following this, the remaining citations were sorted for screening in order of highest to lowest likelihood of relevance, as perceived by the AML tool. The AML tool continued to learn as reviewers screened citations, resulting in re-ordering of citations with time as the classification algorithm became more accurate. Once 95% of the citations predicted to be relevant by the AML tool had been screened and included at Level 1 (454 of 473 predicted relevant citations in 5,665 citations screened), we implemented the AI reviewer in DistillerSR^®^ to act as an independent reviewer to exclude the remaining 10,251 citations (highest remaining relevance probability score = 0.1279). These remaining citations were also screened by a human reviewer, and all conflicts were resolved with the aid of a second human reviewer. This approach aligns with recent guidance for the use of artificial intelligence in title/abstract screening (42).

All citations and full texts were screened in duplicate and independently by a team of seven reviewers (and the AI reviewer at Level 1, as described above). Conflicts were resolved via discussion and arbitration with a third reviewer, if necessary.

### 2.5 Data extraction

Data extraction was performed by two reviewers (DW and BH) in DistillerSR^®^. Data extraction forms were piloted on batches of five studies, with refinements made to the forms as needed, until both reviewers were comfortable with the data to be extracted and conflicts were minimized. Data verification was conducted on 50% of the included references. The following elements were extracted:

- *Publication characteristics*: First author’s last name, year of publication, country of study or first author’s country
- *Study characteristics*: Study objective, data collection methods
- *Demographic data*: Service user subgroup (e.g., people with concurrent mental health conditions, Indigenous people, people of specific gender/sex/sexual orientation subgroups)
- *Study setting data*: Where the interventions were delivered or where the participants were recruited, such as no context (e.g., national or online surveys, online self-help programs), primary care, community-based alcohol treatment services, emergency department, etc.
- *Intervention data*: No specific intervention (i.e., study focused on access to any treatment); interactive non-pharmacologic therapies (e.g., mutual aid groups, psychosocial interventions), plus the mode of delivery (e.g., in person, telemedicine); independent or technology-based therapies (e.g., self-help, web-based programs); pharmacologic therapies, care models (e.g., stepped care), adjuncts to therapy (e.g., body sensors).
- *Barrier/facilitator data*: Modifiable service-level factors reported to be barriers to or facilitators of accessibility to PAU treatment were extracted from the Results section of each paper as they emerged, without an *a priori* list of factors of interest. Concepts were copied and pasted electronically into DistillerSR^®^ or paraphrased, if necessary due to length. As per our eligibility criteria, factors related to the service user, society, or the healthcare system were not extracted; however, factors related to service users’ capacities that correlated with service-level factors were extracted (e.g., an inability to pay was considered a service-level barrier related to the cost of the service). Service provider personality traits, knowledge/training, self-confidence, role responsibility, and the therapeutic relationship were not factors of interest because they were considered to impact treatment success and retention more than accessibility. However, certain service provider characteristics that were reported to be barriers of treatment accessibility were extracted (e.g., language, culture, sex/gender). Where available, we extracted service users’ “preferred levels” that could facilitate treatment accessibility (e.g., preferred appointment times).

Barrier data were coded during data extraction to the most appropriate barrier concept, with new barrier concepts added as they emerged (e.g., “Cost,” “Location,” “Appointment hours,” “Wait period”). Relevant text for each barrier was extracted to provide greater context and depth. Data could be coded to more than one concept; data could be recoded, if necessary, during data verification, cleaning, or synthesis.

Risk of bias assessment was not conducted, as per accepted scoping review methodology (32).

### 2.6 Mapping/synthesizing the evidence

Data were cleaned and collated in Microsoft Excel by DW. Given the scoping review approach undertaken, the objective of our synthesis was to map barrier concepts rather than to synthesize them through a qualitative methodology [e.g., meta-ethnography (43, 44)] or to rank them according to importance. Barrier concepts were mapped by DW to dimensions of Levesque’s Conceptual Framework of Access to Healthcare (39) and verified by an independent reviewer (BH or KT) through discussion to overcome known challenges to the use of the framework (45). Levesque and colleagues defined healthcare access as “the possibility to identify healthcare needs, to seek healthcare services, to reach the healthcare resources, to obtain or use health care services, and to actually be offered services appropriate to the needs for care (39).” Within this framework, “access” is portrayed as an interplay between the “accessibility” of treatment services and the “abilities” (or “capacities,” a term preferred by our research team) of individual treatment seekers throughout the pathway of healthcare utilization (39). “Accessibility” relates to traits of treatment services and is conceptualized by Levesque et al. to have five dimensions: Approachability, Acceptability, Availability and accommodation, Affordability, and Appropriateness. Five corresponding dimensions of the “capacity” of individuals to interact with the accessibility dimensions to obtain access were also conceptualized: Capacity to perceive, Capacity to seek, Capacity to reach, Capacity to pay, and Capacity to engage (39). The focus of this review was on dimensions of accessibility and the service-level factors that they encompass.

After mapping to the Levesque framework, where possible, similar barrier concepts were amalgamated within an accessibility dimension. Synthesis of data from service user subgroups was explored within barrier concepts, where there was sufficient available data. An additional synthesis was conducted that incorporated studies focused on treatment accessibility barriers during the COVID-19 pandemic.

### 2.7 Involvement of people with lived experience and the public

We engaged people with lived experience during the planning and conduct of this research. Input was gathered from multiple organizations as represented in the make-up of our authorship team, to ensure findings are of relevance to multiple groups. Specifically, we engaged with the Community Addictions Peer Support Association, the Canadian Psychological Association, the Canadian Centre on Substance Use and Addiction and the Mental Health Commission of Canada, and we thank them for their valuable contributions to this work.

## 3 Results

### 3.1 Extent of the literature

**Figure 1:**
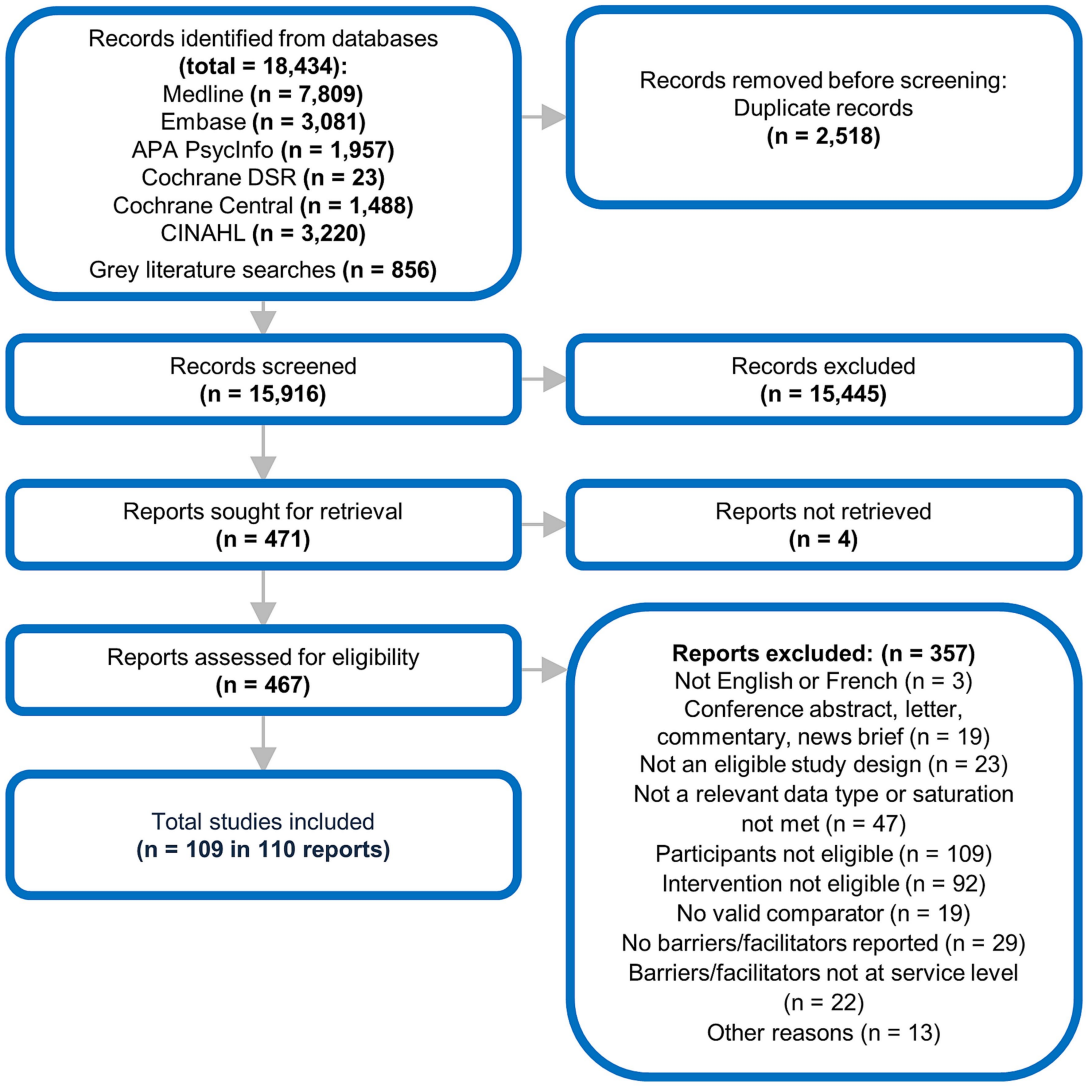
Evidence flow diagram.

Following removal of duplicates, 15,916 references were screened at Level 1, with 471 references included for full-text screening at Level 2. Of these, 109 studies in 110 reports were retained in our review (Figure 1). References excluded at full-text screening (n = 284) and their reasons for exclusion have been provided in Supplementary Table 4.

### 3.2 Study characteristics

**Figure 2:**
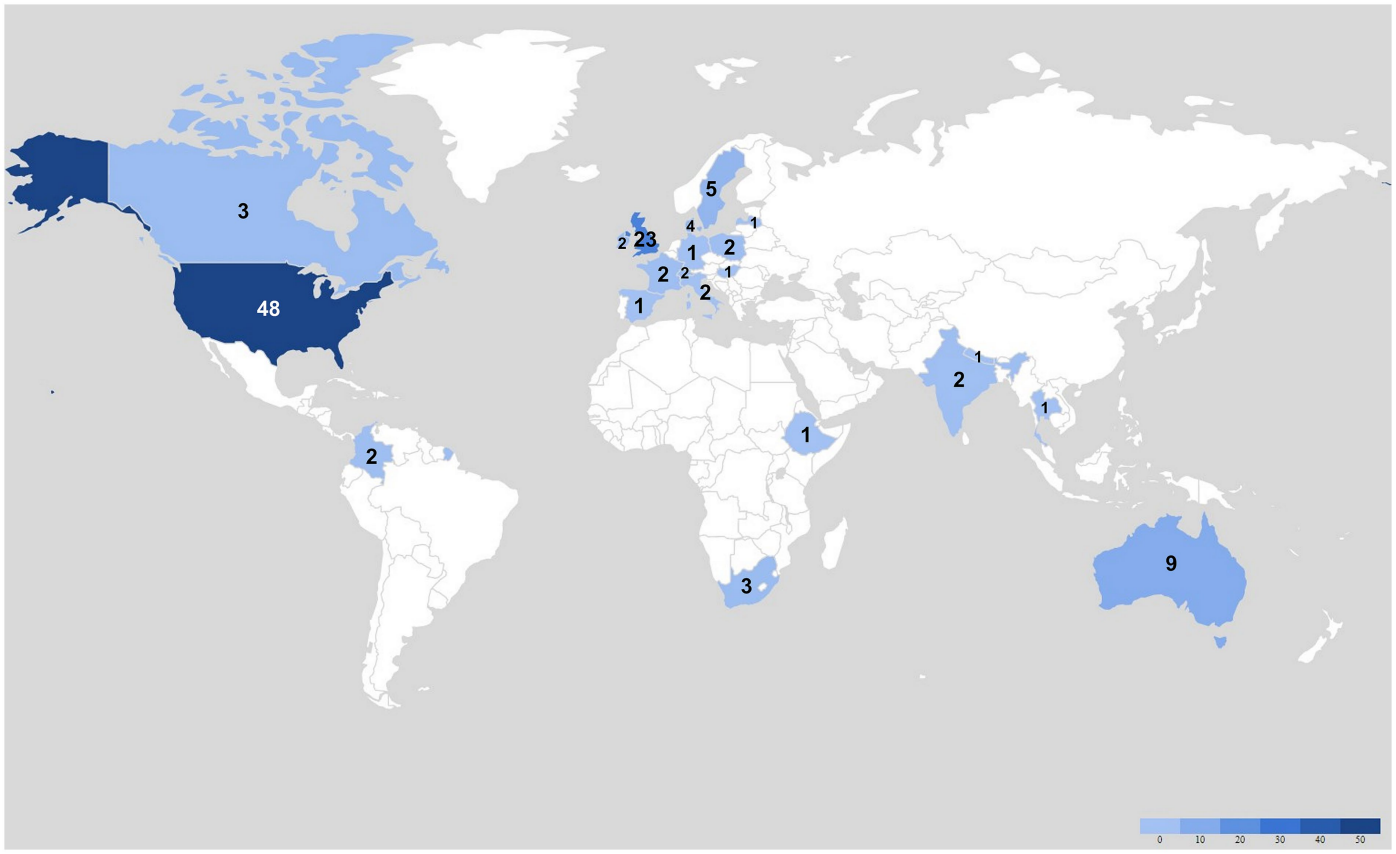
Countries of study conduct.

Characteristics of the 109 included studies are summarized in Tables 1–3, while individual study details are reported in Supplementary Table 5 (with further details in Supplementary Datasheet 1). The geographical distribution and frequency of the included studies are depicted in Figure 2. Years of publication ranged from 2010 to 2022. Forty-nine of the studies (45%) included participants of no specific subgroup (i.e., participants were selected based solely on their PAU status and/or because they were seeking treatment to reduce their alcohol intake) (16, 17, 29, 46–91). Forty studies were not conducted in a specific context (i.e., participants were recruited from the community or online and the intervention had no specific context) (21, 29, 48, 50, 51, 53, 55–58, 63, 64, 66, 81, 86–88, 90, 92–111). Thirty-four studies evaluated no specific intervention (e.g., evaluations of general treatment seeking or engagement) (17, 21, 29, 47, 51, 58, 61, 63, 65, 68, 74, 75, 88, 91, 92, 95, 97, 100–102, 105, 106, 109–120), while 59 studies evaluated some type of screening or non-pharmacologic psychosocial therapy. Independent or technology-based therapies were evaluated by 12 studies (48, 53, 55, 57, 63, 83, 94, 98, 108, 109, 121, 122), and two studies evaluated pharmacologic therapies (extended-release naltrexone (XR-NLT)) (123, 124); two evaluated models of care, including a military-based confidential care model (125) and a primary care collaborative care model (72); and four studies evaluated adjuncts to therapy (e.g., alcohol sensors and monitors, exercise therapy, mindfulness-based therapy) (46, 71, 78, 126, 127). Data collection methods among the 109 studies included interviews only (n = 57) (16–18, 21, 29, 48, 49, 52–56, 61–63, 68, 70–73, 75, 77, 78, 80, 85, 86, 89, 92, 93, 96–99, 101, 103, 105–107, 110, 113–118, 122, 128–139), focus groups only (n = 6) (50, 87, 108, 127, 140, 141), surveys only (n = 26) (46, 47, 57, 64–66, 74, 81, 83, 84, 90, 91, 94, 95, 100, 102, 112, 121, 123, 124, 126, 142–146), and combinations of methods (n = 20) (51, 58, 60, 67, 69, 76, 79, 82, 88, 104, 109, 111, 119, 120, 125, 147–150). Sample sizes ranged from 6 to 1,556 participants (median = 35), with larger studies tending to use survey designs. Three studies reported findings related to PAU telehealth service accessibility during the COVID-19 pandemic in Canada/USA (17), India (16), and the UK (18).

**Table 1:** Summary of the participant characteristics in 109 included studies.

Participant group^b^	Number of studies^a^
General population	49 (16, 17, 29, 46–91)
Sex/gender	17 (21, 93, 101, 102, 107, 109, 110, 118, 127, 131, 135, 139, 144, 145, 148–150)
Sexual orientation	3 (92, 102, 139)
Concurrent physical health conditions	10 (61, 97, 116, 119, 120, 123, 133, 134, 139, 141)
Concurrent mental health conditions	6 (68, 93, 98, 105, 117, 150)
Concurrent mental health conditions and substance use disorders	1 (131)
Indigenous people of the USA or Australia	3 (111, 131, 147)
Other ethno-cultural groups	5 (21, 58, 94, 109, 110, 135)
People who attended emergency departments	5 (113, 128, 136, 143, 146)
Inpatients	5 (99, 113, 126, 130, 149)
Emerging adults	5 (93, 108, 112, 122, 128)
Older adults	3 (18, 104, 137)
Veterans	3 (100, 106, 118)
People who were underhoused	3 (114, 124, 129)
People found to be impaired by alcohol while driving	3 (103, 140, 142)
Concurrent problematic drug use	2 (115, 132)
Military personnel	2 (95, 125)
People with justice system involvement	2 (145, 148)

a Studies could appear in more than one group for each study characteristic.

b Study participant subgroups were determined by study eligibility criteria (e.g., inclusion restricted to a specific subgroup) or by reporting of results specific to a subgroup in a study of a broader population.

**Table 2:** Summary of the contexts of 109 included studies.

Study context^b^	Number of studies^a^
No specific context (community or online sample)	40 (21, 29, 48, 50, 51, 53, 55–58, 63, 64, 66, 81, 83, 86–88, 90–111, 124)
Community alcohol treatment services, including mutual aid groups and other outpatient therapies	16 (46, 49, 52, 54, 61, 62, 68, 71, 73, 74, 76, 78, 84, 85, 89, 123)
Primary care clinics	12 (65, 69, 70, 72, 75, 77, 79, 80, 82, 115, 132, 133)
Inpatient/live-in alcohol treatment	6 (61, 68, 126, 127, 149, 150)
Emergency departments	5 (113, 128, 136, 143, 146)
Non-clinical community services	5 (60, 114, 124, 129, 135)
Hospitals, excluding live-in treatment and emergency departments	4 (113, 117, 130, 141)
Community health services	3 (47, 117, 124)
Community mental health services	3 (16, 139, 144)
Drug and alcohol treatment services	3 (17, 18, 123)
Anti-retroviral treatment outpatient clinics	2 (134, 141)
Colleges and universities	2 (112, 122)
Indigenous healthcare services or communities	2 (131, 147)
Liver transplant units	2 (61, 116)
Pharmacies	2 (59, 67)
Pre-trial detention facilities/post-incarceration	2 (145, 148)
Army installations	1 (125)
Community justice/drug court programs	1 (142)
Hepatology/ gastroenterology/ endoscopy clinics	1 (142)
Mandatory programs for people found to be impaired by alcohol while driving	1 (140)
Older adult clinical services	1 (137)
Pre-operative assessment clinics	1 (138)
Tuberculosis clinics	1 (120)
Veterans Affairs Women’s Health clinics	1 (118)
Workplace settings	1 (121)

a Studies could appear in more than one context group.

b Study context was determined by where participants were recruited or where the intervention was conducted.

**Table 3:** Summary of the intervention characteristics and data collection methods in the 109 included studies.

Intervention	Number of studies^a^
None (i.e., general treatment seeking)	34 (17, 21, 29, 47, 51, 58, 61, 63, 65, 68, 74, 75, 88, 91, 92, 95, 97, 100–102, 105, 106, 109–120)
Screening/SBIRT	18 (59, 60, 67, 70, 77, 79, 115, 121, 130, 132, 133, 135, 137, 138, 142, 143, 146, 147)
Independent or technology-based therapies	12 (48, 53, 55, 57, 63, 83, 94, 98, 108, 109, 121, 122)
12-step or mutual aid groups	10 (50, 54, 56, 62, 81, 90, 96, 129, 144, 145)
Adjuncts to therapy (e.g., alcohol sensors, exercise therapy, mindfulness)	5 (46, 71, 78, 126, 127)
In-person therapy, generally	4 (54, 104, 136, 150)
Community-based alcohol services	6 (18, 49, 52, 76, 89, 99)
Brief interventions not associated with screening	4 (82, 128, 134, 141)
Contingency management	2 (64, 87)
Pharmacotherapy (i.e., extended-release naltrexone)	2 (123, 124)
Models of care (i.e., military-based confidential care model and an integrated collaborative primary care model)	2 (72, 125)
Other interventions	17 (16, 66, 69, 73, 80, 84–86, 93, 103, 107, 122, 131, 139, 140, 148, 149)
Data collection method	
Interviews only	57 (16–18, 21, 29, 48, 49, 52–56, 61–63, 68, 70–73, 75, 77, 78, 80, 85, 86, 89, 92, 93, 96–99, 101, 103, 105–107, 110, 113–118, 122, 128–139)
Survey only	26 (46, 47, 57, 64–66, 74, 81, 83, 84, 90, 91, 94, 95, 100, 102, 112, 121, 123, 124, 126, 142–146)
Mixed methods	20 (51, 58, 60, 67, 69, 76, 79, 82, 88, 104, 109, 111, 119, 120, 125, 147–150)
Focus groups only	6 (50, 87, 108, 127, 140, 141)

a Studies could include more than one intervention.

### 3.3 Accessibility dimensions

**Figure 3:**
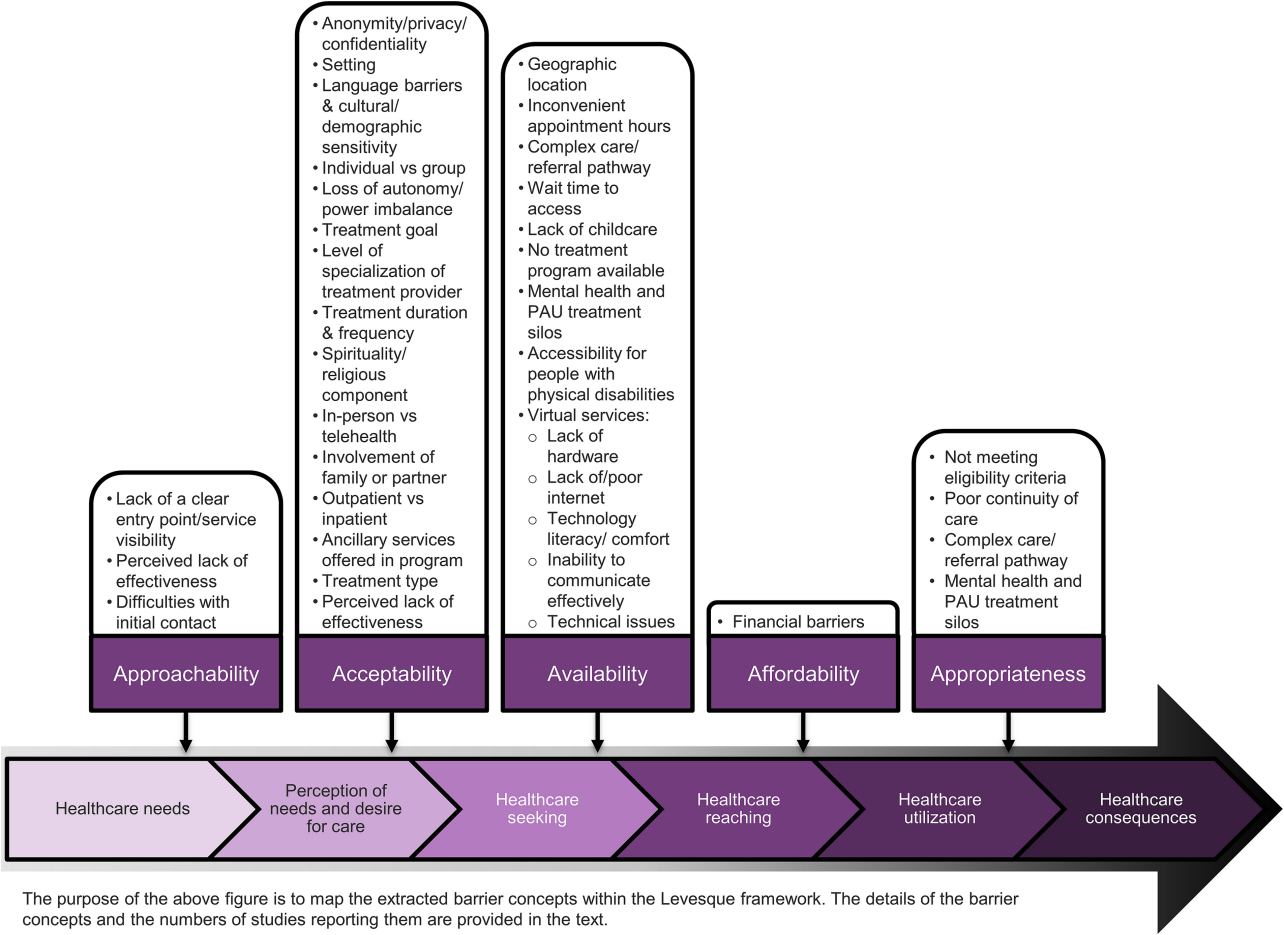
Barrier concepts identified in the literature mapped to the Levesque framework.

The barrier concepts extracted are portrayed in Figure 3, mapped to the accessibility dimensions of the Levesque framework. The richest data were found in the acceptability dimension, which included 20 emergent barrier concepts of which at least one was reported in 84 studies (see Acceptability subsection below). Less rich data were found in the availability/accommodation (14 concepts in 64 studies), approachability (4 concepts in 40 studies), appropriateness (4 concepts in 28 studies), and affordability dimensions (1 concept 25 studies). The most frequently reported barrier concepts were service visibility (acceptability dimension; n = 35 studies), anonymity/privacy/confidentiality (acceptability dimension; n = 32), geographic location and transportation barriers (availability dimension; n = 29), inconvenient or inflexible appointment hours (availability dimension; n = 26), financial barriers (affordability dimension; n = 26), and the complexity of the care/referral pathway (availability and appropriateness dimensions; n = 23). All barrier concepts are described in detail in their respective accessibility dimension below.

#### 3.3.1 Approachability of PAU services

This dimension reflects the capacity of treatment seekers to find and engage with services that they believe can help them (39).

##### 3.3.1.1 Lack of a clear entry point/service visibility (*n* = 33 studies)

Treatment seekers expressed uncertainty regarding where to seek treatment or had sparse knowledge of services available to them. In surveys, not knowing where to go for treatment varied by country, with 49.2% of treatment seekers in Ethiopia (91), 35.8% in Nepal (75), and 8–19% in the USA (58, 101) endorsing it as a barrier to treatment. A greater proportion of men (9%) than women (4%) (101), the majority of people belonging to ethnic minority groups (21, 109–111, 131), and 9.4% of US military personnel with PAU did not know where to seek help (95). Inconsistent dissemination of information about a confidential military treatment program resulted in many military personnel only discovering its existence after self-referring to non-confidential treatment despite potential career implications (125). Similar proportions of people with and without concurrent mental health conditions did not “know where to go” for PAU treatment (100, 105); however, in one study, the majority of Australian Indigenous women with concurrent mental health conditions and SUDs had little knowledge of available services and felt that services were poorly promoted (131). Another study from the UK focusing on specialist community alcohol services reported “marketing and promotion were consistently seen as both important and lacking by service users (76).”

##### 3.3.1.2 Perceived lack of effectiveness (*n* = 14 studies)

The perceived effectiveness of specific PAU interventions influenced treatment engagement for some study participants (21, 29, 47, 54, 66, 88, 98, 100, 104, 105, 110, 111, 124, 136, 145), though extent of endorsement varied substantially across subgroups. In two American survey studies, skepticism about the effectiveness of treatment, generally, was endorsed as a barrier to engagement by 46% of participants with AUD who wanted but did not receive treatment (47), and 75% of urban-dwelling Indigenous American participants felt treatment “would not do any good (111).” Among Americans with and without concurrent mental health conditions, 9.2 and 10.9%, respectively, expressed that they “Did not think anyone could help (105);” however, among US veterans with and without concurrent mental health conditions, 59.1 and 46.3%, respectively, did not think treatment would help (100). Previous unhelpful treatment experienced by themselves (136, 145) or by others (111) dissuaded some from seeking treatment. American Hispanic men generally perceived free treatment as less effective and unaffordable treatment as more effective (110). In a Swedish study, internet- and telephone-based treatments were seen as ineffective (88). Psychotherapy may be preferred over pharmacotherapy by some, including some older adults, because it allows one to understand the causes of problematic drinking (88, 104), whereas pharmacotherapy “merely targets the symptoms without addressing the underlying problems (88).” Low expectations of treatment success were a barrier to engagement with Alcoholics Anonymous (AA) for some American women in pre-trial detention facilities (145), while unrealistically high expectations of therapy may encourage initial engagement in some interventions (e.g., extended-release naltrexone (124) and online self-help (98)).

##### 3.3.1.3 Difficulties with initial contact (*n* = 4 studies)

Some treatment seekers reported difficult initial contact with PAU services as a barrier to engagement (e.g., phone calls unanswered or not returned (29, 99, 117, 150), computer respondent that indicated a two-to-three-month waiting period (150)). Participants expressed feeling dismissed or ignored, and considerable assertiveness was required to obtain treatment (29), although others disengaged (29, 99, 117, 150). “Being able to speak directly to a counselor and having their calls returned was an important factor in not only coming to the treatment center but also in shaping a positive expectation about what they might gain from treatment (150).”

#### 3.3.2 Acceptability of PAU services

The acceptability dimension covers cultural and social aspects of a service that influence a treatment seeker’s willingness to access it (39). We identified a wide range of factors within this dimension, including confidentiality and anonymity, language barriers, discordance in the treatment goals of the treatment seeker and provider, conflicts between a person’s religious or spiritual values and their medical care, restrictions to a person’s involvement in treatment decisions, and provider-patient power dynamics. “Perceived lack of effectiveness” was categorized in both the Acceptability and Approachability dimensions and has been addressed in the Approachability section to avoid redundancy.

##### 3.3.2.1 Anonymity/privacy/confidentiality of service (*n* = 32 studies)

The desire and need for anonymity, privacy, and confidentiality appeared to be driven by fears of stigma and shame (111), deportation (135), and losses of children (53, 58), employment (58, 119, 121), and military careers (95, 125). Concepts related to anonymity, privacy, and confidentiality were frequently found in studies of small or rural communities (56, 89, 111), public non-clinical settings (e.g., pharmacy) (59, 60, 67, 130), the military (95, 125), peer-led groups (56, 62, 70, 74, 99, 111, 145), and text- (108) or internet-based interventions (48, 53, 56, 63, 70, 81, 84, 85, 96, 98). The need for anonymity tended to be highest for those seeking or initiating therapy (62, 63, 96, 108).

##### 3.3.2.2 Setting (*n* = 19 studies)

The treatment setting could negatively influence treatment engagement through perceived stigmatization, triggering fear, first impressions of the condition of the treatment facility, and other factors. Some people with PAU felt stigmatized when treatment was offered in the same location as other drug services (49, 76) or human immunodeficiency virus (HIV) treatment (134); outpatient treatment at a hospital was more acceptable because they could be there for many reasons other than therapy for their PAU (99). Treatment facilities that appeared unsafe (99, 102, 104, 111) or were unfamiliar (136) also discouraged engagement, and locating services in safe (150) or familiar settings (67, 115, 135) may improve accessibility. Interventions set in the ED were preferred by many rather than waiting until the shock of an alcohol-related incident had dissipated (128); however, 30% of participants in the ED in another study were too ill to participate (143). In group settings, heterogeneity of participants with respect to treatment goal (e.g., abstinence or consumption reduction) (136) or reason for treatment (i.e., people with an alcohol-impaired driving conviction forced to attend AA) (140) could negatively impact engagement.

##### 3.3.2.3 Language barriers and cultural/demographic sensitivity (*n* = 7 studies)

Lack of care sensitive to a person’s own ethno-cultural group or in the language of their choice was an accessibility barrier identified by Indigenous people (111, 131), other ethno-cultural groups (21, 58, 66, 107, 110, 135), people living with deafness (89), older adults (104), military personnel (106, 118), and people of different sexual orientations (102). When linguistic or cultural disconnects are present, service providers may be perceived as not fully understanding service users’ experiences (104, 106, 107, 110, 135), including the social norms of drinking within their culture (107, 135). People of cultural minority groups may also feel that service providers of the cultural majority are judgmental (107) or invasive (110). In peer-led groups of mixed cultures, people of cultural minorities may feel singled out (111) but feel comfortable in groups of people of their own culture (111, 151). Women also may prefer women-only groups due to content and style of communication (144), safety (144, 150), and the potential for men to trigger underlying mental health conditions (118).

##### 3.3.2.4 Individual vs. group treatment (*n* = 17 studies)

Preference for individual or group therapy varied across participants within studies, potentially limiting accessibility, if the preferred treatment format was unavailable (53, 61, 89, 118, 120, 136, 145). Some who did not like to be singled out preferred a group format (53, 120), and many derived support from exchanging stories (29, 62, 119). Online mutual-aid groups were appreciated by those who enjoyed the flexibility of accessing support anytime and anywhere (62). However, groups were perceived negatively by some because of the lack of individualized attention (97, 110), triggering of social anxiety (99, 119), concerns about confidentiality (99, 119), focusing on other people’s problems (51), focus of sessions being devoted to those who were still drinking (99), and a preference to be in groups of people who were abstinent (119). Some people felt that group and individual therapy had differing purposes: group therapy supported recovery, whereas individual therapy was seen to deal with complex psychological issues (29), and treatment availability in concordance with their perceived needs may encourage engagement. Specifically, individual treatment provided an individualized connection for some that helped to maintain a sense of personal responsibility (17).

##### 3.3.2.5 Loss of autonomy and power imbalance (*n* = 15 studies)

Many treatment seekers feared losing control of their lives with traditional prescribed “police-style” PAU interventions (53, 54, 63, 88, 111, 114) or with AA’s concepts of powerlessness and peer authority (54), causing some to access self-paced online treatment (53). Patient-centered care that involved participants in personal healthcare decision making (29, 68, 72, 85, 103) and provided flexibility in treatment delivery options (e.g., in-person or telehealth) (72) promoted feelings of empowerment, responsibility, and independence (54, 85). However, some people with PAU have preferred having less autonomy if they became overwhelmed by the onus of responsibility of treatment seeking when access to treatment is extremely difficult (29), or if therapist supervision would force them to engage more and be more accurate in reporting their alcohol consumption (53).

Power imbalances that negatively impacted treatment engagement were identified in group treatment settings, including the perceived paternalistic sponsorship model of AA and some other mutual aid groups (54, 73, 111), the authoritarian indoctrination of 12-step groups (73), and the abstinence hierarchy perceived in groups of people at all stages of recovery (104, 114). In other settings, power imbalances were found in the rules and reminders to follow in live-in treatment (150) and the need to comply with PAU treatment from a general practitioner (GP) who also provides individuals with methadone for their other substance use problems (115).

##### 3.3.2.6 Treatment goal (abstinence vs. consumption reduction) (*n* = 16 studies)

Treatment engagement was reduced when disconnects existed between the treatment goal of a program (i.e., abstinence vs. consumption reduction) and the goal of the treatment seeker. When faced with forced abstinence, some did not enroll because they felt the treatment goal was too difficult to attain or maintain (72, 86, 88, 89, 104), and some opted out because they felt pressured to conform (99) or were antagonized by abstinent group members for preferring consumption reduction (96). However, for others, an abstinence commitment may make them feel a part of a group (96) and reinforce their self-esteem when met (86). Consumption reduction as a goal was preferred by many (79, 88) and was perceived as motivating and positive (104) and more easily attainable (86), providing an intermediate goal toward abstinence (54, 86). In some areas, there was a perceived gap in service provision for people wanting to moderate their drinking rather than abstain (63). Some programs appeared to support consumption reduction in principle, only to expel participants that perceived moderation to be an end and not an intermediate goal toward abstinence (29). Programs supporting both abstinence and consumption reduction goals were generally appreciated (49, 72, 86, 104); however, some seeking abstinence feel that such programs enable drinking (72).

##### 3.3.2.7 Level of specialization of treatment provider (*n* = 14 studies)

The level of specialization of the provider of community-based PAU counselling influenced some treatment seekers’ engagement; some preferred to access treatment with their primary care clinic and others preferred a specialist. Treatment within primary care clinics may be preferred by many over specialist clinics (65, 72, 79, 115, 147) because of the pre-existing therapeutic relationship (79), the convenience and low cost (72), and primary care being perceived as a less drastic and stigmatizing step to take (88). Within primary care, in one study, nurses were preferred over doctors for screening and brief intervention (SBI) (79), but there was no preference between doctors and either computers (146) or telephone-based interactive voice recognition (77). Others preferred to access specialist PAU therapy due to perceptions that expert knowledge was needed to treat PAU (88) and primary care may not be able to access all diagnostic or treatment modalities (69). Regarding inpatient treatment, some accessing PAU treatment in general inpatient settings felt that their symptoms were often minimized and that they were not as accepted as they would be in a specialty inpatient program (88). However, some with complex mental health conditions appreciated general over specialist inpatient services (68). Finally, most pharmacy users found SBI acceptable, although a minority suggested that they preferred to discuss alcohol with their primary care doctor (59, 67).

##### 3.3.2.8 Treatment duration and frequency (*n* = 9 studies)

The duration of a treatment program, whether too short or too long, impacted program engagement, depending on the treatment seeker’s perceived needs and abilities (53, 89, 108, 118, 128, 133, 134). Preferred program duration often varied among study participants (53, 89, 108, 118, 128, 134), with those with a history of higher-risk drinking perceiving programs as too short, while those with a history of lower-risk drinking perceiving programs as adequate or too long (134). Similarly, the frequency of therapy could impact engagement (29, 68, 89), with the preferred frequency varying across treatment seekers: high frequency of therapy required too great of a time commitment for some (68, 89), while low frequency of therapy potentially offered insufficient support for others (29).

##### 3.3.2.9 Spiritual/religious component (*n* = 8 studies)

Spiritual or religious aspects of some PAU interventions were perceived negatively (53, 54, 68, 98, 114, 145, 150), depending on service users’ personal beliefs. Many perceived AA to have an oppressive approach, decreeing that participants surrender to a Higher Power and rely on belief rather than objective reasoning (54). Many participants expressed that such approaches were incompatible with their beliefs, even those who were religious or spiritual (54), or were offensive or triggering for those who were not (54, 150). Some chose secular online (53, 98) or in-person (54) alternatives to AA to separate recovery from religion.

##### 3.3.2.10 In-person vs. telehealth (*n* = 6 studies)

In-person therapy was preferred over telehealth by service users in several studies (18, 76, 84), while preferences were mixed in others (17, 85). In-person face-to-face therapy felt “more genuine, more personable,” a better therapeutic relationship was developed, there was the potential for deeper conversations and to read body language, and it was easier to follow or to ask for help than by video (84, 85), telephone (18), or either virtual delivery method (17). As well, people using telehealth services may not be able to access the full suite of therapies that are provided through in-person care (76). Despite this potential limitation, telehealth minimized the need for some service users to cancel appointments (85), provided greater flexibility in timing (17, 18, 85), reduced the anxiety of initial engagement in group therapy (18), and enabled access during COVID lockdowns (16).

##### 3.3.2.11 Involvement of partner or family (*n* = 4 studies)

Participants in some studies suggested that they would be more likely to engage in PAU treatment if a support person (e.g., spouse/partner, children, caregiver) could also attend (50, 116, 120, 137) to give them a better understanding of PAU, treatment, and how to foster abstinence (116). This concept was found mainly in studies of population subgroups such as people with concurrent alcohol-related liver disease/transplant (116) or tuberculosis (120) and older adults (137).

##### 3.3.2.12 Outpatient vs. inpatient (*n* = 4 studies)

Many treatment seekers felt that inpatient therapy was “a last resort” and preferred outpatient to inpatient therapy (88, 92, 102), with a greater proportion of bisexual compared to heterosexual individuals being afraid of being placed in hospital if they sought treatment (92). For people with PAU who were underhoused, inpatient treatment offered a sense of stability and a time-out from “the hardships of life on the streets and from the 24-h job of obtaining, drinking, and recovering from the effects of alcohol (114).” However, inpatient therapy wasn’t seen as an effective form of therapy to maintain abstinence (114).

##### 3.3.2.13 Ancillary services offered within the treatment program (*n* = 2 studies)

Service users in two studies suggested that they would be more likely to engage with PAU treatment if ancillary services were also offered, such as life-skills/employment training (68, 136) or free access to a gym (136).

##### 3.3.2.14 Treatment type (*n* = 21 studies)

Numerous studies reported service user preferences for different types of treatments that have not been reported above. Barriers to treatment engagement could occur when treatment seekers could not access their preferred treatment [e.g., only AA available but preferred another intervention (97, 110, 111)]. A summary of participant treatment type preferences has been provided in Supplementary Table 7.

#### 3.3.3 Availability of PAU services

The availability dimension suggests that PAU services are accessible if they can be reached where and when they are needed by the treatment seeker (39).

##### 3.3.3.1 Geographic location and transportation barriers

The geographic location of PAU services or lack of transportation were common barriers to treatment accessibility (17, 18, 50, 53, 54, 58, 66, 74, 76, 85, 89, 95, 99–102, 105, 111, 119, 123, 126, 131, 136, 145, 150), especially for people living in rural areas or outside of the urban core (18, 76, 85, 89, 99, 131), people with physical disabilities (123, 136), and people with concurrent mental health conditions (100). Some preferred telehealth or internet-based treatment services because they overcame geographical considerations, transportation issues and logistical hassles, and were timesaving (17, 53, 85). Receiving PAU treatment from an existing service that people with PAU already attended (e.g., primary care clinic, liver transplant program, non-clinical community services) was preferred by many because of convenience (72, 116, 135).

##### 3.3.3.2 Inconvenient or inflexible appointment hours (*n* = 26 studies)

Inconvenient or inflexible appointment hours were reported to be a barrier to accessibility by participants in many studies (17, 18, 29, 48, 53, 58, 66, 76, 85, 87, 89, 91, 92, 95, 100–102, 105, 111, 115, 125, 131, 133, 134, 141, 145), especially those who were employed (53, 66, 76, 85, 87, 89, 95, 100, 102, 111, 125, 133, 134). Telehealth options provided greater flexibility in timing and, therefore, greater accessibility (17, 18, 48, 53, 85). For people with PAU and concurrent mental health conditions, inflexible service hours resulted in poor accessibility at times of crisis (131). Military personnel appreciated appointments outside of duty hours because they were free to attend in civilian clothes, they did not need to ask superiors for time off (and so the treatment could remain off their record), and off-duty treatment occupied a time when they were more likely to drink, providing a distraction (125).

##### 3.3.3.3 Complexity of the care/referral pathway (*n* = 22 studies)

Lack of communication, coordination, and agreements between care providers (e.g., hospital and community services, primary care and specialists) resulted in fragmented, time-consuming, and sometimes non-linear referral pathways that contributed to difficulties in navigation and accessibility for service users (21, 29, 51, 68, 69, 74, 75, 88, 89, 97, 104, 110, 113, 150). As well, referral providers’ lack of knowledge of or communication of the existence of available treatment options (54, 89, 118, 150), lack of provision of referral contact information to service users (118), and lack of scheduling of initial appointments for service users (117, 118) were expressed by service users as negatively affecting treatment accessibility. Integrated PAU treatment programming (e.g., within primary care) (72), outreach (76, 148) and in-reach (e.g., “locating potential service users [in hospital] and engaging them prior to formal enrolment” into a program) (76), the use of third-sector charities to aid service-user navigation (106); identification and coordination of services, teams, and clinicians (68, 135); and proactive scheduling of initial appointments for service users by referral providers (103, 113, 118) were all suggested as facilitators of referral pathway navigation and accessibility.

##### 3.3.3.4 Wait time To access treatment (*n* = 17 studies)

The time between identification of a suitable PAU service (or referral to one) and initiation of treatment was a frequently cited barrier to treatment accessibility (21, 49, 58, 68, 74, 76, 89, 97, 101, 103–105, 110, 111, 113, 131, 136, 150). When wait times were prolonged, treatment seekers’ readiness to change could fade (89, 104, 111). Some who felt the need for immediate treatment presented at hospital if they were unable to wait for outpatient therapy (113, 150). However, if they presented in crisis with alcohol toxicity, their help-seeking was sometimes dismissed as hospital staff perceived them not to be serious about recovery (150). Services were suggested to be overburdened (21, 110) due to staff shortages (49) or funding cuts (76), exacerbating wait times.

##### 3.3.3.5 Lack of childcare (*n* = 10 studies)

Lack of childcare was a barrier to PAU treatment that varied by country and subgroup. In general population surveys in the USA, “Could not arrange for child care” was endorsed infrequently as a barrier to accessing PAU treatment (0–2%) (58, 101, 105). However, surveys of Nepalese (66) and Ethiopian people (91) with AUD found 58 and 22%, respectively, reported problems with childcare as a barrier to PAU treatment. In specific US subpopulations, such as veterans (100) and Indigenous people (111), 10–30% expressed that difficulty obtaining childcare was a PAU treatment barrier. Among US veterans, women were significantly more likely than men to report lack of childcare as a PAU treatment barrier (26% vs. 18%), as were people with a concurrent mental health condition compared to those without (29% vs. 11%) (100). In US general population surveys, no differences in the proportion endorsing childcare as a barrier were found between US ethnic groups (58); however, event counts were too low to detect a difference between women and men (101) and those with and without concurrent mental health conditions (105). Online treatment programs were easier to engage in for some treatment seekers because they did not have to obtain childcare during the time they engaged with treatment (53).

##### 3.3.3.6 No treatment program available (*n* = 8 studies)

An overt lack of existing treatment programs or interventions was identified as a barrier to PAU treatment, especially for those in rural areas (89, 99) and those seeking specific interventions, such as treatment integrated into primary care (80, 104), tuberculosis clinics (120), or liver transplant programs (116), or alcohol-specific treatment for people with opioid use disorder (OUD) (115). A minority of military personnel reported that there were no providers in their community, preventing them from obtaining treatment (95).

##### 3.3.3.7 Mental health and PAU treatment silos (*n* = 5 studies)

Lack of treatment programs that offered both mental health and PAU interventions was reported as barrier in 5 of 7 studies that included people with concurrent mental health conditions (68, 76, 99, 117, 131). Siloed mental health and PAU services requiring abstinence to receive mental health therapy and stable mental health to receive PAU treatment resulted in people with concurrent disorders not being accepted for either program (68, 76, 99, 131). Some felt that the combined services available to them did not meet their individual needs, with one participant stating, “my psychiatrist’s way of looking at it was just to abstain from alcohol, all problems will be resolved (117).”

##### 3.3.3.8 Accessibility for physically disabled (*n* = 2 studies)

People with PAU and concurrent physical disabilities reported that in-person treatment accessibility was impeded by lack of wheelchair lifts or platforms (89) and that those who could not physically use a computer could not access computer-based screening and treatment services (70).

##### 3.3.3.9 Technology-based therapy: lack of hardware (*n* = 5 studies; published 2014–20)

For some attempting to access technology-based therapy, such as telehealth video appointments, text-messaging services, or mobile phone-based apps, engagement was contingent upon owning a computer or a mobile phone (17, 18, 55, 69, 94). Provision of a mobile phone by the service provider ensured accessibility to treatment in one study (55).

##### 3.3.3.10 Technology-based therapy: lack of or poor internet (*n* = 5 studies; published 2012–22)

Not having access to the internet was a barrier to telehealth video appointments reported by older adults (18). Poor or unstable internet also forced some people with PAU to reject telehealth as a treatment option over in-person therapy (17, 85). Similarly, some could not complete computer based SBIs in venues such as the ED or primary care that had unstable internet (70, 143).

##### 3.3.3.11 Technology-based therapy: low technology literacy or comfort (*n* = 8 studies; published 2010–22)

Technology-based therapies such as telehealth by videoconference, computer- or tablet-based PAU screening, and mobile phone apps were inaccessible for some due to low technology literacy or being uncomfortable with technical devices (17, 18, 69, 84, 85, 137, 142, 146). Subgroups that appeared to have low technology literacy included older adults (18, 69, 137), those with learning disabilities (18), and those with lower education or experience with computers in rural areas (69) or among people found impaired with alcohol while driving who were most vulnerable to unmet treatment needs (142). Some preferred not to engage with telehealth by videoconference despite being computer literate because they already spent enough time on the computer (84).

##### 3.3.3.12 Technology-based therapy: inability to communicate effectively (*n* = 3 studies; published 2017–21)

Some found engagement with interventions delivered by email, telephone, or videoconference difficult (18, 57, 85). Weekly email guidance for an internet-based CBT program was difficult as a form of conversation (57). Older adults had difficulties both hearing and communicating via telehealth by phone, especially those with memory problems, speech or hearing impairments, or serious mental health issues (18). Videoconference was reported to be a poor medium to provide CBT, when cognitive exercises were drawn on a blackboard that could not be seen properly (85).

##### 3.3.3.13 Technology-based therapy: technical issues (*n* = 3 studies; published 2013–22)

Minor and major technical issues could prevent engagement with computer-assisted SBIRT (143), cellular photo digital breathalyzers (71), and any telehealth intervention (17).

#### 3.3.4 Affordability of PAU services

This dimension reflects a treatment seeker’s capacity to spend money and time on services. It results from direct service-associated costs and opportunity costs associated with lost income (39).

##### 3.3.4.1 Financial barriers (*n* = 25 studies)

The out-of-pocket cost—determined by the amount charged by the service provider, non-treatment costs such as transportation, and the amount offset by any insurance coverage held by the service user—was found to be a substantial barrier to service accessibility in a large number of studies (*n* = 26) (21, 47, 58, 64, 66, 72, 75, 81, 91, 92, 95, 97, 100–102, 104, 105, 110, 111, 118–120, 126, 135, 141, 150). In US population-based surveys, 8–20% of respondents reported that they “could not afford to pay the bill” or that “health insurance did not cover it (58, 101, 105),” with people with concurrent mood or anxiety disorders being significantly more likely to express financial barriers to care compared to people without (19.2% vs. 9.8), at least in part attributable to differential insurance coverage between the groups (30.9% vs. 15.9% without insurance, respectively) (105). In US subpopulations, the proportions of people with PAU reporting financial barriers were higher than the general population for veterans (56.3% with concurrent mental health conditions and 45.7% without) (100), Indigenous people (55.4% could not afford and 51.8% had no insurance) (111), and people in the Hispanic community (21, 110), many of whom did not have health insurance due to employment or legal status (135). However, for people using services funded by the Health Resources and Services Administration (HRSA), low proportions expressed financial reasons as a barrier to treatment (2%) (47), and those using an online mutual aid group (81) and a collaborative care model based in primary care (72) found accessibility facilitated by affordable services. Financial barriers in other countries varied, endorsed by 4% of people surveyed in Italy, Germany, Hungary, Latvia, Poland, and Spain (75), 42.2% in Ethiopia (91), and 96.5% in Nepal (66).

Wide variations in insurance coverage drove inequalities in treatment accessibility. In Canada, a two-tiered healthcare system reduced treatment accessibility for those who relied on programs covered by government insurance: those willing to pay out of pocket could access more timely, longer, and more specialized treatment than that offered by insurance-covered programs (150). Similarly, some US Indigenous people felt that insurance would only cover a limited duration of treatment that was inadequate to their needs, and to go elsewhere was too expensive (111). Hispanic men in the USA felt that effective treatment was reserved for those who could afford it or were insured, and that the poor or uninsured could only access inadequate treatment (21, 110).

#### 3.3.5 Appropriateness of PAU services

The appropriateness dimension refers to how well a service meets a treatment seeker’s needs, how quickly it is delivered, how thoroughly health problems are evaluated and treatments selected, and how high a service’s technical and interpersonal standards are (39). The complexity of the care/referral pathway and mental health and PAU treatment silos were also categorized in the Availability dimension and have been covered there.

##### 3.3.5.1 Not meeting eligibility criteria (*n* = 11 studies)

Treatment seekers suggested that they had not met PAU treatment program eligibility criteria based on condition severity (29, 70, 99), capacity to achieve and sustain abstinence (104), readiness to change (150), immigration status (110), and stable mental health (68, 76, 99, 131), and that sometimes eligibility assessments had been incorrect (e.g., level of severity or readiness to change were misinterpreted) (99, 150). Some treatment seekers felt that PAU treatment was for those who were “really, really ill” or when all else fails (29), or that services were for people who had lost employment, relationships, and/or housing (99), and they therefore assumed that they did not meet eligibility criteria (29, 99).

##### 3.3.5.2 Poor continuity of care (*n* = 6 studies)

Poor continuity of care within a treatment service negatively impacts the therapeutic relationship between service user and provider, and people with PAU may be less likely to engage with services with known poor continuity of care (29, 68, 104, 114). Staff turnover (29, 68, 104, 114) and inconsistency in providers between different phases of treatment (e.g., screening by a nurse followed by BI from a physician) (93, 138) disrupted existing therapeutic relationships.

### 3.4 Pau telehealth service accessibility during the COVID-19 pandemic

Three studies reported findings related to PAU telehealth service accessibility during the COVID-19 pandemic in Canada/USA (17), India (16), and the UK (18).

In Canada and the USA, virtual care resources, including online peer-support groups and individual telehealth psychosocial treatment, were embraced because in the process of reducing the risk of coronavirus transmission, they also eliminated the logistical hassles of driving to and from meetings or appointments and mutual aid groups could be accessed any time of the day or night in different locations around the world (17). Some service users felt that online mutual aid groups allowed them to connect and form relationships with others more easily; however, others expressed that online groups provided less meaningful conversations and made forming personal connections more difficult, preferring to have in-person meetings (17). Virtual services were difficult for some to access due to lack of awareness of the services in the community, lack of hardware or internet access, low technological literacy, and technical problems during sessions (17). Although group sessions were helpful toward recovery, some stated that they would appreciate having individual support options during the pandemic to help them maintain a sense of personal responsibility and because “they missed the individualized connection (17).”

In India, 35% of people with AUD sought treatment via telehealth phone calls compared to 2.6% of people with OUD, indicating possibly that those with AUD were less concerned about privacy issues (16). Unwillingness to use telehealth services was endorsed by a low proportion of people with SUDs (11%) (16).

In the UK, older adults with PAU found telehealth appointments by phone to be highly accessible and appreciated not having to travel to appointments, especially those in remote rural areas (18). Telehealth by phone offered greater flexibility in appointment hours, more frequent contact, and was perceived to have greater anonymity than in-person options (18). However, in-person options were preferred by some service users, given the ease of communication compared to the phone: in-person felt more genuine, more personable, easier to follow, and easier to ask for help and to express feelings (18). No participants in the study used telehealth by videoconference during the pandemic, some due to lack of hardware or access to the internet, some due to technological literacy, and others due to health issues or learning difficulties that prevented them from using technology effectively (18).

### 3.5 Facilitators of service accessibility and service-specific factors

Service users in some studies suggested approaches that could be adopted to overcome service-level barriers and facilitate service accessibility. These facilitators have been summarized in detail by barrier concept in Supplementary Table 8. Briefly, facilitators mainly reflected the barriers reported above, including the following:

- *Approachability*: participants suggested various media to increase the visibility of PAU services in different contexts and some commented that engagement with a counsellor initially by phone was instrumental in accessing a service.
- *Acceptability*: participants highlighted factors that increased anonymity (e.g., online services) and endorsed treatment providers/groups with similar demographics or culture to their own, involvement of family or supportive people in treatment, involvement in their own treatment decisions, and choice regarding treatment goal, level of specialization of treatment provider, and spiritual aspects of programs.
- *Availability*: participants valued telemedicine and treatment embedded into existing services that they regularly attended, flexible appointment hours, and help to navigate complex care pathways.
- *Affordability*: free treatment, low-fee online programs, and treatment embedded in primary care were considered to facilitate accessibility.
- *Appropriateness*: maintenance of continuity of care ensured trust between treatment provider and seeker, facilitating care.

Also, some studies focused on specific interventions for which barriers and facilitators were not generalizable to other PAU services (e.g., screening, pharmacotherapy, online programs or apps). These findings have been summarized in Supplementary Table 9.

## 4 Discussion

To our knowledge, this is the first scoping review to map recent literature regarding service-level barriers to and facilitators of accessibility of PAU treatment services from the perspective of treatment seekers and service users. Our review found numerous unique barriers that were classified to the Levesque framework dimensions of approachability, acceptability, availability, affordability, and appropriateness of PAU services. However, it should be noted that barriers to PAU treatment accessibility do not exist in isolation and multiple barriers from different dimensions may interact to decrease accessibility. For example, one of the barriers that was endorsed across a variety of subpopulations and contexts was that PAU treatment seekers did not know where to go: there was no readily available “door” through which one could access PAU services (Approachability). To find that access point, sometimes multiple disconnected doors must be identified and passed through before access to PAU treatment can be reached, if it’s geographically reachable (Availability). Ultimately, upon gaining access to PAU treatment, treatment seekers may discover that they do not meet eligibility criteria because they do not drink heavily enough, they have a concurrent mental health condition, or they have differing goals compared to those of the program (Appropriateness). Or they may find that the wait period is months long (Availability), the treatment is unaffordable (Affordability), or the program is not culturally appropriate (Acceptability). In being turned away, they may not be directed to a more appropriate treatment service, or that service may not exist. As the final door closes, they have nowhere to go.

As the above example illustrates, several barriers may act together to prevent treatment engagement, and the importance of any given barrier in the treatment seeking process may be difficult to discern. The objective of this scoping review was to map PAU treatment accessibility barriers and not to rank them in order of importance. Although tempting, ranking barriers across studies by vote counting (i.e., inferring that barriers reported more frequently in the literature are of greater importance than those reported less frequently) may be biased by the research questions, populations, contexts, survey tools, and years of conduct of the available primary research studies. The distributions of these study characteristics impact the frequency at which specific barriers may be reported. For example, a few studies (6%) in our review included people with PAU and concurrent mental health conditions. Accordingly, barriers related to concurrent mental health conditions were reported infrequently, including the segregation of mental health services and PAU treatment services, and the inability to meet eligibility criteria requiring the absence of co-existing mental health conditions. Given that the prevalence of co-occurring mental health conditions in people living with PAU is high (152), if vote counting were used, the importance of these barriers would be undervalued. Consequently, we have descriptively summarized the data, attributing equal weight to each barrier.

### 4.1 Contextualizing findings with other reviews

The service-level barriers to PAU treatment accessibility found in this review generally aligned with barriers identified in other recent systematic, scoping, and narrative reviews of the general SUD literature (153–160), some of which have focused on specific subgroups related to female sex or gender (154, 156, 157, 159) or specific treatment attributes (e.g., treatment goal (160), telehealth mode of delivery (153)).

A recent overview of reviews explored barriers and facilitators to SUD treatment (19). Many of the structural barriers to SUD treatment reported in that review (19) demonstrated a congruence with the PAU treatment barriers in our review, including lengthy waiting periods, lack of suitable services for people with concurrent mental health disorders, treatment affordability, lack of connectivity of referral pathway, lack of gender-suitable treatment, and issues with treatment goal and intensity. Lack of confidentiality, privacy, or anonymity was a frequently endorsed service-level barrier in the studies included in our review that caused fears of stigma (111), deportation (135), and losses of parental rights (53, 58), employment (58, 119, 121), and careers (95, 125). The previous overview of reviews classified these concerns about privacy and fears of stigma and loss of child custody as individual-level barriers (19); however, we considered these barriers as potentially modifiable service barriers. Similarly, we considered a lack of transportation to be a service-level barrier related to the geographic location of the treatment service. In the Levesque framework (39), the five dimensions of accessibility are countered by five corresponding dimensions of capacities of treatment seekers, with which they interact to generate access. Therefore, the service location is balanced by the treatment seeker’s capacity to reach the service, which may lead to inequities in access, if the service is located off public transit or in another city. Offering a treatment service in a more convenient location (i.e., modifying the service-level barrier) may improve access for treatment seekers with limited means of transportation.

Regarding subgroups related to female sex or gender, the accessibility barriers to SUD treatment among pregnant people and mothers reported in two reviews (154, 156) were similar to many in our review, including fear of loss of custody, lack of childcare, or childcare as a competing priority. Many sex-related barriers found in our review were amplified in a scoping review of studies of female veterans for whom mental healthcare in the predominantly male military environment was perceived as unwelcoming and insensitive, necessitating a sex-specific approach (157). Integrated gender-responsive approaches have also been advocated for women with SUD and concurrent mental health disorders to overcome many of the difficulties to treatment engagement faced by this very vulnerable group (159).

Two recent reviews reported barriers to treatment accessibility resulting from specific treatment attributes: non-abstinence approaches (160) and a telehealth delivery model (153). Our review found that abstinence-based PAU treatment may pose a barrier to initial engagement, with consumption reduction approaches being preferred by some treatment seekers. Although consumption reduction approaches have been proposed in the SUD treatment literature, limited empirical research has tested the hypothesis that offering goal choice earlier in the treatment process may increase treatment engagement (160). Barriers to virtual care, including unfamiliarity with technology, lack of internet access or hardware, and communication difficulties via telehealth that were endorsed in our review were also identified in a scoping review of barriers to virtual care in the health system, generally (153).

### 4.2 Impact of COVID-19 on PAU treatment accessibility

Globally, the availability of PAU treatment services was impacted by COVID-19 and associated public health restrictions (5–10); however, only three studies met our review eligibility criteria regarding COVID-19 impacts. Temporal constraints may account, in part, for the paucity of COVID-19 literature identified, as pandemic-related delays in the conduct and peer review of primary research may have precluded publication prior to our April 2022 search date. Additionally, our review eligibility requirement of service-level barriers meant that studies reporting only policy-level barriers related to the pandemic more broadly, such as lockdowns (7, 161), were ineligible for inclusion. Strategies to mitigate the effects of COVID-19 by substance use treatment facilities early in the pandemic were reported in a scoping review (12). That review identified several mechanisms by which substance use treatment services attempted to support service users, including telehealth, use of remote monitoring devices, altering prescription practices and distribution of medications, and extension or adjustment of appointment hours (12). However, despite attempts to facilitate substance use treatment service accessibility, COVID-19 restrictions and control measures ultimately acted to decrease the availability of care and service capacity by reducing employee working hours, appointment hours, care visits, live-in care beds, and group treatment offerings (12).

### 4.3 Strengths and limitations of the review methods and the available literature

We used rigorous scoping review methods to identify and synthesize data on a diverse and challenging topic in a replicable and transparent manner. Using a well-established framework, we distilled a large volume of rich qualitative data into a coherent narrative. We optimized qualitative validity and reliability through the use of strict eligibility criteria, a codebook of barriers, open discussion during data extraction/verification, and triangulation of data from multiple studies during synthesis (162). However, as in other studies that have used the Levesque framework (39, 45), our ability to categorize barriers to accessibility dimensions was sometimes limited by the level of detail of the barriers reported in the included studies. Few studies were conducted in low-to-middle-income countries and a large proportion were conducted in the USA, meaning that the findings from the review may have low generalizability outside of high-income countries. Finally, manuscript length prevented full presentation of the extracted data, and we encourage readers to view our Supplementary data sheets.

### 4.4 Strengths and limitations of the research question

We posed a highly focused research question that concentrated on service-level barriers and did not include person- or societal-level barriers to treatment. This allowed for greater attention to service-level factors that could be modified but did not provide a broad view of the barrier landscape, where cultural drinking norms, lack of awareness or acceptance of the severity of a problem (4, 19), and societal stigma substantively impact initiation of treatment seeking. A variety of sources of stigma are known to negatively influence treatment seeking, including structural stigma, stigma held by service providers, stigma in the community, and self-stigma. While we acknowledge these sources of stigma, they were not considered to be service-level factors and, therefore, were beyond the scope of our review. We also acknowledge that service provider factors, such as knowledge/training, role responsibility, and personality traits (e.g., empathy, self-confidence) can also substantively impact provision of care and retention in care. However, these factors mainly impact treatment accessibility indirectly (e.g., by reducing availability of care), and therefore we elected not to include these factors.

### 4.5 Future directions

Our review findings could be used to support the design and implementation of new treatment services or modification of existing services for people with PAU. The rich qualitative findings from our review will allow future discrete choice experiments (DCEs) to draw on the views of people with PAU, lessening the chance of misspecification of preferred service characteristics as a result of placing too much weight on the opinions of experts or researchers (163). We recognize that barriers to provision of care also negatively impact treatment availability and, therefore, treatment accessibility, and future research should consider this perspective.

## 5 Conclusion

In conclusion, modifiable service-level barriers to the accessibility of PAU treatment are rich in diversity and depth. While many different barrier concepts determined the acceptability of a treatment service from the perspective of people with PAU, the affordability and availability of the service also appeared prominently in primary studies. The results from this scoping review can be used to develop strategies to determine the importance of specific service-level barriers to PAU treatment accessibility within a given context or population, with an ultimate goal of targeted modification of existing treatment services and design of new diverse services to overcome accessibility issues.

## Data availability statement

The original contributions presented in the study are included in the article/Supplementary material, further inquiries can be directed to the corresponding author.

## Author contributions

DW: Conceptualization, Data curation, Formal analysis, Investigation, Methodology, Project administration, Resources, Software, Supervision, Validation, Visualization, Writing – original draft, Writing – review & editing. BH: Conceptualization, Data curation, Formal analysis, Funding acquisition, Investigation, Methodology, Project administration, Resources, Software, Supervision, Validation, Visualization, Writing – original draft, Writing – review & editing. KC: Conceptualization, Funding acquisition, Investigation, Methodology, Validation, Writing – review & editing. NC: Conceptualization, Funding acquisition, Investigation, Methodology, Validation, Writing – review & editing. SNg: Conceptualization, Funding acquisition, Investigation, Methodology, Validation, Writing – review & editing. SNo: Data curation, Methodology, Resources, Validation, Writing – review & editing. JP: Conceptualization, Funding acquisition, Investigation, Methodology, Project administration, Validation, Writing – review & editing. AG: Data curation, Resources, Validation, Writing – review & editing. MD: Data curation, Resources, Validation, Writing – review & editing. AP: Data curation, Resources, Validation, Writing – review & editing. KS: Funding acquisition, Resources, Validation, Writing – review & editing. BS: Methodology, Project administration, Resources, Software, Writing – review & editing. MB: Funding acquisition, Validation, Writing – review & editing. GG: Funding acquisition, Validation, Writing – review & editing. LD: Funding acquisition, Validation, Writing – review & editing. AP: Funding acquisition, Validation, Writing – review & editing. BP: Funding acquisition, Validation, Writing – review & editing. SK: Conceptualization, Validation, Writing – review & editing. AE: Conceptualization, Methodology, Validation, Writing – review & editing. KT: Conceptualization, Data curation, Formal analysis, Funding acquisition, Investigation, Methodology, Project administration, Resources, Supervision, Validation, Visualization, Writing – review & editing.
